# Brain inflammation is induced by co-morbidities and risk factors for stroke

**DOI:** 10.1016/j.bbi.2011.02.008

**Published:** 2011-08

**Authors:** Caroline Drake, Hervé Boutin, Matthew S. Jones, Adam Denes, Barry W. McColl, Johann R. Selvarajah, Sharon Hulme, Rachel F. Georgiou, Rainer Hinz, Alexander Gerhard, Andy Vail, Christian Prenant, Peter Julyan, Renaud Maroy, Gavin Brown, Alison Smigova, Karl Herholz, Michael Kassiou, David Crossman, Sheila Francis, Spencer D. Proctor, James C. Russell, Stephen J. Hopkins, Pippa J. Tyrrell, Nancy J. Rothwell, Stuart M. Allan

**Affiliations:** a Faculty of Life Sciences, University of Manchester, Manchester, UK; b Wolfson Molecular Imaging Centre, University of Manchester, Manchester, UK; c Clinical Neurosciences Group and Stroke Medicine, Salford Royal Foundation Trust, UK; d Health Methodology Research Group, University of Manchester, UK; e North Western Medical Physics, Christie Hospital, Manchester, UK; f SHFJ – CEA Orsay, France; g Brain and Mind Research Institute, University of Sydney, NSW 2050, Australia; h Discipline of Medical Radiation Sciences, University of Sydney, NSW 1825, Australia; i School of Chemistry, University of Sydney, NSW 2006, Australia; j NIHR Biomedical Research Unit, University of Sheffield, Sheffield, UK; k Department of Cardiovascular Science, University of Sheffield, UK; l Metabolic and Cardiovascular Diseases Laboratory, Alberta Institute for Human Nutrition, University of Alberta, Edmonton, Alberta, Canada

**Keywords:** Brain, Co-morbidity, Inflammation, Risk factors, Stroke, Systemic

## Abstract

Chronic systemic inflammatory conditions, such as atherosclerosis, diabetes and obesity are associated with increased risk of stroke, which suggests that systemic inflammation may contribute to the development of stroke in humans. The hypothesis that systemic inflammation may induce brain pathology can be tested in animals, and this was the key objective of the present study. First, we assessed inflammatory changes in the brain in rodent models of chronic, systemic inflammation. PET imaging revealed increased microglia activation in the brain of JCR-LA (corpulent) rats, which develop atherosclerosis and obesity, compared to the control lean strain. Immunostaining against Iba1 confirmed reactive microgliosis in these animals. An atherogenic diet in apolipoprotein E knock-out (ApoE^−/−^) mice induced microglial activation in the brain parenchyma within 8 weeks and increased expression of vascular adhesion molecules. Focal lipid deposition and neuroinflammation in periventricular and cortical areas and profound recruitment of activated myeloid phagocytes, T cells and granulocytes into the choroid plexus were also observed. In a small, preliminary study, patients at risk of stroke (multiple risk factors for stroke, with chronically elevated C-reactive protein, but negative MRI for brain pathology) exhibited increased inflammation in the brain, as indicated by PET imaging. These findings show that brain inflammation occurs in animals, and tentatively in humans, harbouring risk factors for stroke associated with elevated systemic inflammation. Thus a “primed” inflammatory environment in the brain may exist in individuals at risk of stroke and this can be adequately recapitulated in appropriate co-morbid animal models.

## Highlights

► Risk factors for stroke include atherosclerosis, obesity, diabetes and hypertension. ► Stroke risk factors are associated with peripheral inflammation. ► Corpulent rats and atherogenic mice show increased inflammation in the brain. ► Pilot data show that patients at risk of stroke may also develop brain inflammation. ► Chronic peripheral inflammation can drive inflammatory changes in the brain.

## 1 Introduction

Clinical and experimental evidence implicates inflammation in multiple phases of stroke aetiology and pathology (Allan et al., 2005; Amor et al., 2010; Denes et al., 2010a,b; McColl et al., 2009; Muir et al., 2007). Several of the risk factors for stroke, such as atherosclerosis, hypertension and diabetes/obesity are triggered and/or propagated by dysregulated systemic inflammatory processes (Dandona et al., 2004; Ross, 1999; Savoia and Schiffrin, 2006). Markers of elevated systemic inflammation are associated with increased stroke risk and brain lesions detected by magnetic resonance imaging (MRI) (Fornage et al., 2008; van Dijk et al., 2005). Like other statins, rosuvastatin, has multiple anti-inflammatory properties. It reduces cerebrovascular events in patients without hyperlipidemia but with raised C-reactive protein (CRP) levels (Ridker et al., 2008). Angiotensin-converting enzyme (ACE) inhibitors can lower median CRP levels and result in better long-term outcome in stroke patients, after controlling for confounding variables and concomitant treatments (Di Napoli and Papa, 2003). Similarly, aspirin or other anti-platelet treatments are used prophylactically in patients at risk, but it is still unclear whether their beneficial properties are due to anti-aggregation effects or to a combination of anti-platelet and anti-inflammatory effects (Franks et al., 2010). Therefore, although inflammation-driven co-morbidities are common and aetiologically important in stroke patients, exactly how systemic inflammation contributes to risk of stroke and to other neurological conditions remains to be determined.

Despite the almost ubiquitous nature of co-morbidities preceding stroke, there has been a relative paucity of studies incorporating these in experimental stroke research. This may have contributed to the lack of successful translation for a number of potential stroke treatments identified in pre-clinical studies (Endres et al., 2008; Fisher et al., 2009). One reason for the failure of translation may be that underlying inflammation associated with atherosclerotic risk factors modifies the mechanisms of post-ischaemic brain damage, including the type, magnitude and kinetics of the damaging processes. In support of this, we and others have shown that the extent of brain injury is exacerbated, and mechanisms of damage altered and/or aggravated, when experimental stroke is induced in animals with hypertension, diabetes, obesity or acute/chronic infection/inflammation (Coyle, 1984; Denes et al., 2010a,b; McColl et al., 2007; Terao et al., 2008; Vannucci et al., 2001). However, it is unclear whether co-morbid stroke risk factors can drive brain inflammation and induce a “primed” inflammatory state in the brain prior to a cerebrovascular event.

Here we undertook a translational study to determine if risk factors for stroke, which involve chronic systemic inflammation, also induce brain inflammation in rodents and humans. We show that brain inflammation is present in rats and mice harbouring systemic vascular and/or metabolic disease and that analogous changes may be present in patients with clinical risk factors and evidence of systemic inflammation, as indicated by a raised concentration of circulating CRP.

## 2 Materials and methods

### 2.1 Pre-clinical studies

These studies were performed on (JCR:LA-cp) (*cp*/*cp*) corpulent rats, which are obese, atherosclerotic and insulin resistant and ApoE-deficient (ApoE^−/−^) mice fed an atherogenic diet, which exhibit severe atherosclerosis.

Animals were allowed free access to food and water and were maintained under temperature, humidity and light-controlled conditions. All animal procedures adhered to the UK Animals (Scientific Procedures) Act (1986).

Corpulent and lean heterozygous control rats (+/?), obtained from an established breeding colony at The University of Alberta, Edmonton, Canada (Mangat et al., 2007); were subject to PET scanning using specific translocator protein (TSPO; formerly known as peripheral benzodiazepine receptor) radiotracers [^18^F]DPA-714, at 9 (average body weight; +/?: 411 ± 14 g; *cp*/*cp*: 720 ± 22 g), 12 (+/?: 438 ± 18 g; *cp*/*cp*: 918 ± 33 g) (*n* = 4 per group) and 15 months of age (+/?: 452 ± 15 g, *n* = 4; *cp*/*cp*: 0.979 ± 0.054 kg, *n* = 3).

Experiments were carried out in male ApoE^−/−^ (JAX 2052, Jackson Laboratories, USA) and C57BL/6 control mice (Jackson Laboratories, USA) bred in-house at the University of Sheffield. Mice aged 8 weeks were fed normal chow (4.3% fat, 0.02% cholesterol) or a high fat/high cholate (Paigen; 18.5% fat, 0.9% cholesterol, 0.5% cholate, 0.26% sodium) diet (Special Diet Services, UK) for 8 weeks.

#### 2.1.1 Positron emission tomography

Rats were anaesthetised by isoflurane inhalation (induction, 5%; maintenance, 2–2.5%) in oxygen. [^18^F]DPA-714, a specific tracer for the TSPO (Boutin et al., 2008; Chauveau et al., 2009) was synthesised (James et al., 2008), and injected intravenously in the tail vein as a bolus (10.8–19.8 MBq, 0.03–2.79 nmol). Respiration and temperature was monitored throughout using a pressure sensitive pad and rectal probe, Model 1025L interface and PC-SAM software (SA Instruments, NJ, USA). Body temperature was maintained at 37 ± 0.5 °C by use of a heating pad and the heating and fan module connected to the rectal probe via the interface and controlled by the PC-SAM software. Whole-body images were acquired in list-mode with a non-rotating 16-module quad-HIDAC PET camera (Oxford Positron Systems, UK) for 1 h (Hastings et al., 2007). The list-mode data were reconstructed directly into 5 min time-frame images (without resorting to histogramming) via the one-pass-list-mode-expectation maximisation (OPL-EM) algorithm (Reader et al., 2002) with one iteration of 16 sub-sets into images of dimensions 120^2^ (transaxially) × 240 (axially) with isotropic 1 mm^3^ voxels. Absolute calibration of the images was achieved by reference to a [^22^Na] source imaged in the field of view in each scan. This had been validated with a uniformly filled mouse-sized [^18^F] phantom imaged over 2 h. Dynamic images were calibrated in kBq/cm^−3^.

Images were segmented using the Local Means Analysis method and the organ mean Time Activity Curves were corrected for Partial Volume Effect using the Geometric Transfer Matrix (GTM) method with a selection of 20% of the organ voxels (GTM20) (Maroy et al., 2008a,b). The segmentation method extracts regions with homogeneous TACs, as required by the GTM20 method. The latter was designed to be more robust than the original GTM method to segmentation errors through the automated selection of adequate voxels in the segmented organs. Both methods were applied using the BrainVisa and Anatomist framework. For more accurate quantification and illustration purposes, PET images were co-registered with the rat MRI template (Schwarz et al., 2006), generously provided by GlaxoSmithKline (Verona, Italy). Automatic segmentation of PET images revealed 1–2 regions of interest (ROI) with different [^18^F]DPA-714 (low and high) uptake in the brain of both the lean and corpulent rats. These ROIs were used to compare the genotypes and the different ages.

To account for the differences (∼2-fold) in body-weight between lean and corpulent rats, we expressed all uptake values as standardised uptake value (SUV) (i.e. percentage of injected dose per cubic centimetre corrected for body weight: %ID · kg/cm^3^). Until now the problem of comparing obese and lean animals or patients, and using SUV, has been mainly applied to [^18^F]fluoro-deoxy-glucose PET imaging. However, considering the controversial literature on SUV, and the fact it has been reported that correcting for the absolute body-weight was likely to over-compensate for the difference (Boellaard, 2009; Keyes, 1995; Sugawara et al., 1999) we have used a slightly different approach. Indeed, the over-compensation of SUV is due to the fact that it assumes that the excess of weight mainly due to adipose tissue has the same metabolic activity than the rest of the body (Keyes, 1995; Sugawara et al., 1999), and therefore that corpulent rats have a metabolic activity twice that observed in lean controls. To the contrary, we considered that correcting for the lean body-weight was likely to under-compensate since it assumes that the excess of adipose tissue is completely inert (Keyes, 1995). Both assumptions being wrong, we decided to adjust the body-weight to calculate the SUV according to Kleiber laws (Kleiber, 1947), in which the metabolic activity is proportional to a factor equal to *m*^0.74^ (*m* being the body-weight in g of the animal).

#### 2.1.2 Tissue processing

Under terminal anesthesia, mice and rats were perfused transcardially with saline followed by 4% paraformaldehyde (PFA; Sigma, UK). Brains were removed and postfixed in 4% PFA at 4 °C for 24 h. Brains were subjected to cryoprotection in phosphate-buffered saline containing 20% sucrose for 24 h. Five alternate sets of 20 μm (mice) or 30 μm (rats) thick coronal brain sections were cut on a sledge microtome (Bright series 8000; Bright Instruments, Huntingdon, UK). All sections were collected into an antifreeze solution (containing 30% ethylene glycol (Sigma, UK) and 20% glycerol (Fisher, UK) in phosphate-buffered saline) and stored at −20 °C until processing.

#### 2.1.3 Immunohistochemistry

Immunohistochemistry was performed on free-floating brain sections. Endogenous peroxidise activity was blocked with 0.3% hydrogen peroxide (Sigma) in dH_2_O and sections were treated with 2% normal serum (Vector Laboratories, Burlingame, CA) for 1 h at room temperature. Sections were incubated overnight in antibody diluent (0.1 M PBS + 0.3 % Triton X-100, Sigma) using the following primary antibodies: goat anti-mouse VCAM-1 1:250 (R&D Systems, UK), goat anti-mouse ICAM-1 1:250 (R&D Systems, UK), goat anti-mouse Iba1 1:500 (Abcam, UK), rabbit anti-Iba1 (Wako Chemicals, Germany) and rat anti-mouse CD45 1:250 (Serotec, UK). Sections were then incubated in appropriate biotinylated secondary antibody for 1 h (rabbit anti-goat 1:1000 and rabbit anti-rat 1:750, Vector Laboratories, UK). Sections were then incubated in Vectastain ABC solution (Vector laboratories, UK) and colour was developed by nickel enhanced diaminobenzidine (50 mg/ml) incubation (Vector Laboratories, UK). Sections were mounted onto gelatine coated slides, dehydrated and coverslipped using Depex (Fisher, UK). Images were collected on an Axiocam colour CCD camera (Zeiss, Germany) upright microscope using 20× and 60× objectives and captured using a Coolsnap ES camera (Photometrics) through Axiovision software (Zeiss, Germany).

#### 2.1.4 Immunofluorescence

Double or triple immunofluorescence was performed on free-floating brain sections. After blocking in 2% normal donkey serum (Vector Laboratories) sections were incubated overnight at 4 °C in primary antibodies: rat anti-mouse CD45 1:200 (Serotec, UK), goat anti-mouse VCAM-1 1:250 (R&D Systems), goat anti-mouse ICAM-1 1:250 (R&D Systems), rat anti-CD3 (Serotec), goat anti-Iba1 (Abcam, UK), rabbit anti-Iba1 (Wako Chemicals, Germany) and rabbit anti-neutrophil serum (SJC), kindly provided by Drs. Daniel Anthony and Sandra Campbell, University of Oxford (Anthony et al., 1998). The antigens were visualised with the adequate fluorochrome-conjugated (Alexa 594 1:750 or Alexa 488 1:500, Molecular Probes) secondary donkey antisera or with biotinylated secondary antibodies followed by streptavidin Alexa 350 conjugate, for 2 h at room temperature. Sections were mounted onto gelatin-coated slides and cover-slipped Vectashield mounting medium containing diamidinophenylindole (Vector Laboratories, Burlingame, CA).

Images were collected on an Olympus BX51 upright microscope using 40× and 60× objectives and captured using a Coolsnap ES camera (Photometrics, UK) through MetaVue Software (Molecular Devices, UK). Specific band pass filter sets for DAPI, FITC and Texas red were used to prevent bleed through from one channel to the next.

#### 2.1.5 Quantitative analysis

All quantitative analysis was performed under blinded conditions and confirmed by at least two independent researchers. VCAM-positive blood vessels were counted in three random fields of view for each section (typically 8–10) containing rostro-caudal cerebral cortex. A score for the whole brain was obtained by averaging individual counts and this was expressed as positive blood vessels per mm^2^.

Activated microglia were identified as showing: (1) increased Iba1 immunopositivity, (2) enlarged and/or amoeboid cell body, (3) complete or partial loss of thin, elongated processes. Round shaped, small Iba1-positive cells with leucocyte morphology were not counted. Regions analysed for microglial activation were also stained with mouse anti-rat CD68 (corpulent rats) and rat anti-mouse CD45 (mice) to assess the number of parenchymal macrophages and other leucocytes. Activated microglia were counted throughout the striatum and expressed as activated microglia per mm^2^.

Fluorescently labelled CD45 positive cells were counted in two randomly selected fields of view of the caudal choroid plexus (−1.82 mm from Bregma) and the lateral ventricle (−1.58 mm from Bregma). The choroid plexus and ventricular ependyma were visualised by using VCAM immunofluorescence.

#### 2.1.6 Histology

After CD45 immunohistochemistry (see above) sections were rinsed in dH_2_O and incubated in 60% v/v isopropanol/dH_2_O (Fischer, UK) for 2 min. Sections were transferred to Oil red O (ORO; Sigma, UK) (0.05% w/v ORO/99% isopropanol) for 15 min, rinsed in 60% v/v isopronanol, rinsed in dH_2_O and coverslipped with an aqueous glycerol jelly mount (7.7% w/v Gelatine (BDH, UK) and 54% glycerol in water). Haematoxylin & Eosin (H&E) staining was performed on mounted brain sections. Following staining sections were dehydrated and cover-slipped with Depex mounting medium.

#### 2.1.7 Statistical analysis

Quantitative analysis of data was performed in a blinded manner. PET image quantifications were analysed using Mann–Whitney for comparison between lean and corpulent animals and for comparing 9 vs 15 and 9 vs 12 month age groups. Because the same group of animals was scanned at 12 and 15 months of age, a non-parametric paired Wilcoxon test was used to compare these two groups.

Quantitative data from immunohistochemical and immunofluorescence studies were analysed by one- or two-way analysis of variance (ANOVA) followed by *post-hoc* Bonferroni’s correction. All data are expressed as mean ± SD. Statistical significance is reported at the 0.05 level.

### 2.2 Clinical study

#### 2.2.1 Patients

This small, preliminary study was undertaken to assess cerebral inflammation in humans with multiple risk factors for stroke, but no evidence of cerebral damage, in order to investigate the relevance of our experimental findings in a translational context. One hundred and twenty-one subjects were screened, and rigorous criteria were applied to identify patients at risk of stroke, while excluding patients with existing brain pathology. Subjects were deemed eligible if having multiple (three or more) risk factors for stroke, and/or established arterial disease (hypertension, dyslipidemia, atrial fibrillation, left ventricular hypertrophy, ischaemic heart disease, diabetes mellitus, peripheral vascular disease, carotid disease and smoking), and CRP >3 mg/L on two separate occasions. All subjects underwent MRI scans to exclude any intracranial pathology, and subjects with a history of a previous cerebrovascular event were not involved in the study. MR scans were reviewed by neuroradiologists. Only four patients fulfilled all inclusion criteria and were subjected to PET imaging to assess microglial activation in the brain (see below). Age matched control participants were chosen on the basis of having two or fewer major vascular risk factors and plasma CRP ⩽1 mg/L (see Table 1). All participants were also screened to exclude cognitive impairment and a telephone consultation was used to exclude symptoms of acute infection prior to PET scanning. All participants gave written informed consent.

#### 2.2.2 Positron emission tomography

Participants underwent MRI scans on a 3 T Philips Achieva system using a T1 weighted inversion recovery SENSE sequence for co-registration of PET images and to exclude visible evidence of stroke. PET studies were performed on a high resolution research tomograph (CTI/Siemens). [^11^C](R)-PK11195 (TSPO ligand) was used to assess microglial activation in the brain. Following a 6 min transmission scan, [^11^C](R)-PK11195 was injected as a slow bolus over 20 s and data were acquired during a 60 min emission scan. The injected radioactivity dose was 465 ± 121 MBq and radiochemical purity was always greater than 98.9%. The injected mass of cold (R)-PK11195 was 2.4 ± 1.0 μg. Binding potential (BP_ND_) images were generated using the simplified reference tissue model and a supervised clustering algorithm was used to extract a reference tissue input function (Turkheimer et al., 2007). The study was approved by the local research and ethics committee.

## 3 Results

### 3.1 PET imaging reveals neuroinflammation in *cp*/*cp* JCR-LA cp rats

**Fig. 1:**
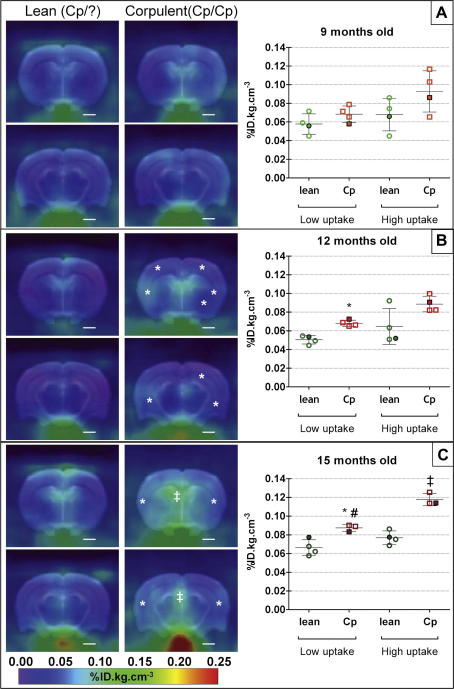
Sum images (20–60 min post-injection; left panel) and respective quantification (graphs on the right panel) of [^18^F]DPA-714 uptake in the brain of lean (+/?) and corpulent (*cp*/*cp*) rats at 9 (A), 12 (B) and 15 months (C) of age. * and ‡ indicate a significant difference between lean and corpulent animals of the same age in respectively low and high uptake regions of interest (*P* < 0.05, Mann–Whitney test). # indicates a significant difference between 9 (A) and 15 (B) months old animals (*P* < 0.05, Mann–Whitney test). Data are expressed as and mean ± SD (filled symbols correspond to the respective image on the left panel).

There was no significant difference in microglial activation as determined by PET imaging between lean and corpulent rats at 9 months of age (Fig. 1A). By 12 months of age, microglial activation was increased significantly in the brains of the corpulent rats in the ROI with the lowest tracer uptake (+35%, Fig. 1B). [^18^F]DPA-714 uptake increased further in 15 month old animals (+32% and +53% in low and high uptake ROI respectively; Fig. 1C). We also observed a trend for an increase in neuroinflammation with age in both lean and corpulent animals, although this was significant only in the corpulent in the low uptake ROI when comparing 15 with 9 month old animals (+28%, *P* < 0.05, Fig. 1C). Although the [^18^F]DPA-714 uptake was increased by a similar magnitude (+29 to 33%, Fig. 1B and C) between 12 and 15 months in the corpulent rats, the differences were not significant. In reference organs, known to express high level of TSPO (heart, lungs and kidneys), there were no significant differences between lean and corpulent rats (Supplementary Fig. 1).

### 3.2 Immunohistochemical evidence of microglial activation in rodents with peripheral disease

**Fig. 2:**
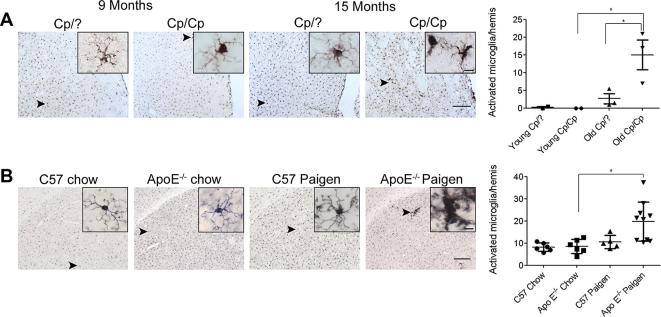
Rodent models of atherosclerosis involve microglial activation in the brain. (A) Activated microglia as identified by increased Iba1 immunopositivity, thickened processes and irregular cell bodies were seen in the striatum of 15 month old corpulent rats, but not in 9 month old animals. Aged corpulent rats had a significantly increased number of activated microglia compared to young corpulent, or 15 month old heterozygous rats. (B) Activated, Iba1-positive microglia was numerous in ApoE^−/−^ mice fed a Paigen diet. Insets show representative images of microglial cells from the different groups of mice. Quantitative analysis revealed significantly more activated microglial cells in the striatum of ApoE^−/−^ mice fed a Paigen diet compared with ApoE^−/−^ mice fed chow diet. ^∗^*P* < 0.05. Scale bars: 200 and 10 μm (insets).

Immunohistochemistry revealed activated microglial cells in the brains of 15 month old corpulent rats (Fig. 2A). We found no activated microglial cells in the brains of corpulent rats aged 9 months or in heterozygous (lean) rats at any age (9–15 months) examined.

In ApoE^−/−^ mice fed Paigen diet Iba1 immunohistochemistry revealed activated microglial cells (Fig. 2B). Microglia displaying thickened processes and increased levels of Iba1 were observed in multiple brain regions such as the cerebral cortex, striatum, hypothalamus, periventricular areas and meninges. Chow or Paigen-diet fed C57BL/6 control mice and chow-fed ApoE^−/−^ mice lacked activated brain microglia.

### 3.3 Atherogenic mice develop vascular inflammation and leucocyte infiltration in the brain

**Fig. 3:**
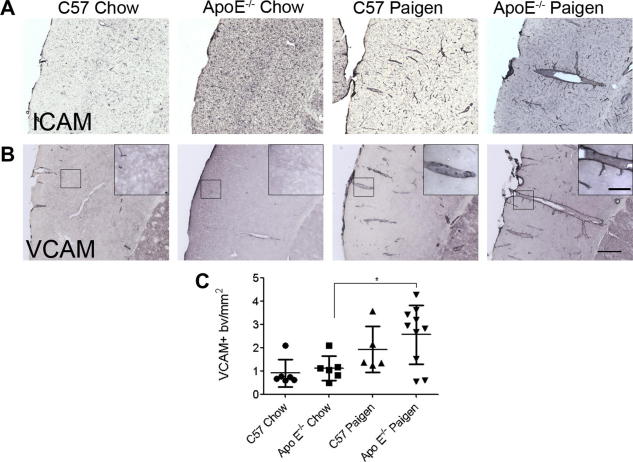
Cerebrovascular activation occurs in the brain in association with peripheral atherosclerosis. Vascular activation was assessed in the cerebral cortex using immunostaining to the adhesion molecules (A) ICAM and (B) VCAM. Unlike mice fed a chow diet, mice fed a Paigen diet showed an increased number of ICAM and VCAM-positive blood vessels in the brain. (C) Quantitative analysis of VCAM-positive blood vessels in the cerebral cortex. Scale bars: 200 and 50 μm (inset).

C57BL/6 or ApoE^−/−^ mice fed a chow diet did not show elevated vascular ICAM or VCAM expression in the brain. A trend for increased vascular ICAM and VCAM expression was observed in C57BL/6 mice fed the Paigen diet (not significant). In contrast, ICAM and VCAM expression was significantly augmented in ApoE^−/−^ mice on the Paigen diet (Fig. 3A and B). Increased VCAM staining was present mainly on medium sized or large blood vessels in the cerebral cortex, striatum, thalamus and hippocampus. Quantitative analysis of VCAM immunohistochemistry revealed significantly stronger staining in Paigen fed groups compared to chow diet (*P* < 0.01, data not shown). Post-hoc comparison revealed significant differences between ApoE^−/−^ chow and Paigen fed animals (Fig. 3C), but not in C57BL/6 mice, indicating that diet-induced pro-inflammatory changes are augmented in ApoE^−/−^ mice.

**Fig. 4:**
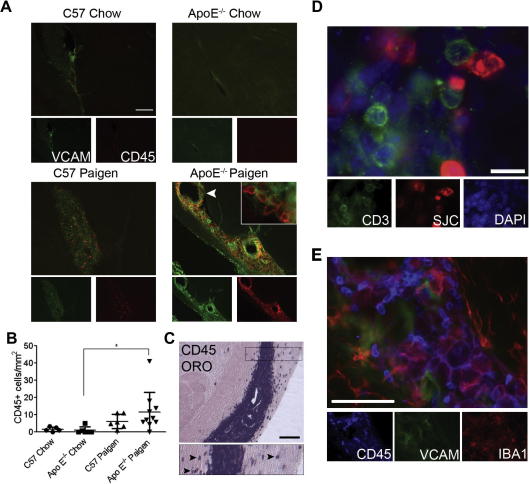
Microglia/macrophages, granulocytes and T cells accumulate in the choroid plexus of the caudal lateral ventricle in response to peripheral atherosclerosis. (A) ApoE^−/−^ mice fed a Paigen diet show accumulation of CD45^+^ leucocytes (red) in the choroid plexus of the caudal lateral ventricle, which display increased VCAM (green) immunopositivity. (B) Quantification of CD45^+^ leucocytes in the choroid plexus of the lateral ventricles. (C) CD45-positive cells, which are numerous in the choroid plexus, also appear in the parenchyma (Oil red O counterstain) on both sides of the lateral ventricle (inset, arrowheads). (D) CD3 positive T cells (green) were found to accumulate in a partially overlapping area with granulocytes, identified with an anti-neutrophil serum (SJC, red). (E) A population of microglia/macrophages (Iba1, red) shows increased CD45 immunopositivity (blue) in the caudal choroid plexus among other CD45-positive leucocytes (possibly granulocytes). ^∗^*P* < 0.05. Scale bars: A; 200 μm; C; 100 μm; D; 10 μm and E; 50 μm.

We also investigated the possibility that diet-induced atherosclerosis was associated with leucocyte infiltration into the brain parenchyma and ventricles, using immunofluorescent staining of the common leucocyte antigen CD45. Microglial CD45 expression was relatively dim throughout the brain and was well discriminated from that of bright and round shaped or elongated leucocytes. Profound enrichment of ventricular leucocytes was found in ApoE^−/−^ mice fed with Paigen diet, and this was associated with increased VCAM expression in the choroid plexus (Fig. 4A). Invasion of the choroid plexus by CD45-positive cells was significantly elevated in ApoE^−/−^ animals on the Paigen diet compared to ApoE^−/−^ animals on normal diet, but this was not observed in C57BL/6 mice fed with Paigen diet (Fig. 4B). In Paigen-fed ApoE^−/−^ mice, CD45-positive cells were numerous in the choroid plexus of the lateral ventricles from the *fimbria hippocampi* to the caudal areas of the ventricle. Caudally, infiltration of ventricular-associated cells into the surrounding parenchyma was also observed in ApoE^−/−^ mice (Fig. 4C). The size of the lateral ventricle was not significantly different among experimental groups and no correlation between CD45-positive cells and ventricle size was found in individual mice. The choroid plexus was found to contain a number of different cell types including granulocytes (identified by an anti-neutrophil serum, SJC) and CD3-positive T cells (Fig. 4D). Granulocytes represented a large proportion of the cells and were uniformly distributed along the VCAM-positive areas of the choroid plexus, in partial overlap with T cells. Activated microglia/macrophages lined the walls of the caudal lateral ventricle, showed increased CD45 expression (Fig. 4E).

### 3.4 Atherogenic diet results in focal lipid deposition and inflammation in ApoE^−/−^ mice

**Fig. 5:**
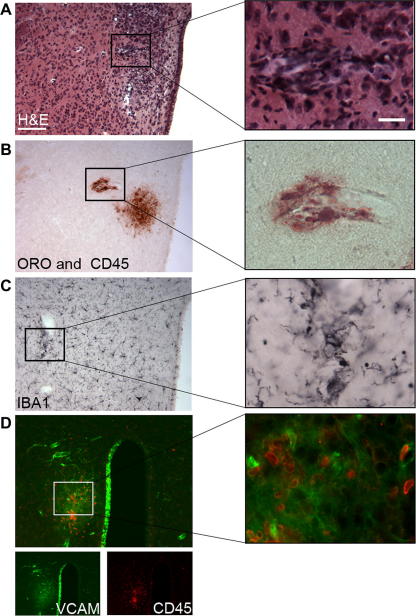
Focal pathological changes are present in the brain in response to peripheral atherosclerosis. Haematoxylin & Eosin (H&E) staining (A) reveals vascular inflammation as indicated by dilated blood vessels and inflammatory infiltrates in the hypothalamus adjacent to the third ventricle in Paigen fed ApoE^−/−^ mice. Focal lipid deposition as identified by Oil red O staining is observed in the vicinity of perivascular CD45-positive leucocytes (B). This is associated with an increase in the number of activated, Iba1-positive microglia (C) recruitment of CD45^+^ cells (D, red) and focally upregulated VCAM immunostaining (D, green). VCAM expression is also seen in the ipsilateral wall of the third ventricle but not in the contralateral part. Parallel brain sections from a representative brain are shown. ^∗^*P* < 0.05. Scale bars: 100 and 12.5 μm. (For interpretation of the references to colour in this figure legend, the reader is referred to the web version of this paper.)

In peripheral tissues, particularly in large blood vessels, ApoE^−/−^ mice develop atherosclerotic plaques, as identified by lipid deposition, leucocyte infiltration and vascular stenosis (Stoll and Bendszus, 2006; Zadelaar et al., 2007). However, it is not known whether such focal vascular pathologies appear in the brain in these animals or not. In 40% of the Paigen fed ApoE^−/−^ mice, focal pathologies were observed in the brain parenchyma (typically in the hypothalamus, near the third ventricle). Oil red staining identified blood vessel-associated lipid deposition (Fig. 5A and B), accompanied by microglial activation and leucocyte recruitment (identified by H&E), as well as CD45 and Iba1 staining (Fig. 5C). VCAM expression was increased focally around lipid rich areas and also in the ipsilateral wall of the third ventricle, indicating ongoing inflammatory responses in the brain (Fig. 5D).

### 3.5 PET imaging: pilot study reveals neuroinflammation in human subjects with risk factors for stroke

**Fig. 6:**
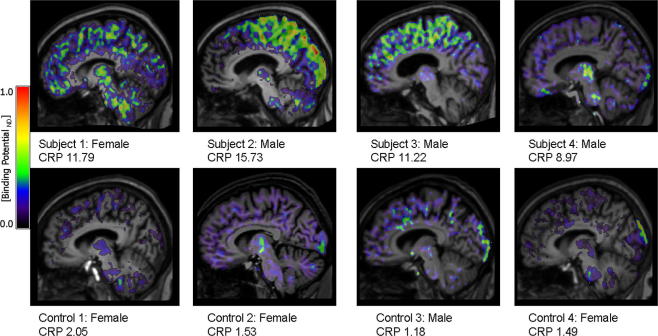
[^11^C](R)-PK11195 binding potential (BP_ND_) images are shown for all subjects and control participants. Images are displayed on each subject’s respective T1 MRI scan normalised to the SPM5 T1 brain template. The value for each individual’s CRP at the time of PET scanning is also shown.

Peripheral inflammatory markers increased in both groups of subjects between screening and time of PET but remained higher in the at risk group (Table 1). Visual inspection of the participants’ BP_ND_ maps revealed increased [^11^C](R)-PK11195 binding in three of the subjects with increased risk factors (Fig. 6). The distribution of the [^11^C](R)-PK11195 signal showed individual differences and was seen across neocortical areas and other brain regions, including the thalamus and brain stem. There was no evidence of raised [^11^C](R)-PK11195 binding in periventricular or deep white matter regions. There was no pattern of activity in a particular vascular territory, as one might see with established stroke. These preliminary results indicate that neocortical inflammation is present in the brain of subjects with chronic systemic inflammation, which is consistent with our findings in rodents with risk factors for stroke.

## 4 Discussion

Here we show that major risk factors for stroke such as atherosclerosis, hyperlipidemia and obesity, which involve chronic systemic inflammation, are associated with brain inflammation in relevant animal models and in a small cohort of humans, in the absence of any cerebrovascular events. These data suggest that systemic inflammation can drive brain inflammation prior to stroke presentation, leading to a “primed” inflammatory environment in the brain.

We used PET imaging to identify microglial activation, because these cells are early responders to pathological changes in the CNS and microglial activation is a hallmark of multiple brain diseases in patients and rodent models (Hanisch and Kettenmann, 2007; Teeling and Perry, 2009). The advantage of assessing neuroinflammation by *in vivo* PET imaging in rodents is that these measurements are comparable with clinical imaging data, and is therefore highly translatable to clinical settings. Both [^11^C]PK11195 and [^18^F]DPA-714 bind to TSPO, but despite that [^18^F]DPA-714 has the advantages of better signal to noise ratio (Chauveau et al., 2009) and the longer half-life of [^18^F], which allow PET imaging of 2–3 animal per batch of tracer, [^18^F]DPA-714 is not yet available for clinical use in our facilities. Corpulent rats exhibited focal areas of microglial activation, as assessed by increased [^18^F]DPA-714 binding *in vivo*. Increased TSPO-ligand binding was observed in various brain areas, including periventricular regions and some subcortical and cortical regions in the corpulent rats (Fig. 1B and C). Imaging data correlated well with the immunohistochemistry findings, which revealed an increase in the number of activated microglial cells. In line with the experimental data, the presence of [^11^C](R)-PK11195 binding indicated neuroinflammation in subjects with multiple risk factors.

Microglial activation was also detected in several brain regions in atherosclerotic ApoE^−/−^ mice, indicating that neuroinflammation is likely to be a common link among animal models of chronic systemic inflammatory diseases. Although the exact mechanism of microglial activation needs to be further investigated, such “priming” of microglia in response to peripheral inflammatory changes has important implications to multiple cerebrovascular diseases. It is now established that microglia primed by central neurodegeneration or amyloidosis respond more vigorously to subsequent systemic or central inflammatory insults. For example, in a murine model of prion disease, intracerebral or systemic LPS challenge induced augmented microglial activation and cytokine expression compared to control mice (Cunningham et al., 2005). Our data indicate that systemic influences are also capable of priming the inflammatory response of the brain. The presence of activated microglia and cerebrovascular inflammation may not only lead to irreversible neuroinflammatory alterations in the brain, but probably contribute to outcome if an ischaemic event occurs. Given that the vast majority of experimental stroke studies are undertaken in ‘normal’ animals with no underlying inflammation this might explain the lack of translation of potential treatments to the clinic.

We performed further characterisation of the vascular and cellular response in the brain of C57BL/6 and ApoE^−/−^ mice, to explore the possible effects of atherogenic diet on neuroinflammation. Although the atherogenic “Paigen” diet alone reportedly induces inflammation in peripheral organs (Desai et al., 2008), we found significant vascular activation or enrichment of CD45-positive cells in the choroid plexus only in ApoE^−/−^ mice fed the Paigen diet, not in C57BL/6 mice. No sign of intraluminal plaques was observed in cerebral blood vessels in our study, which is in line with a report showing increased oxidative stress and endothelial dysfunction in cerebral arterioles in high-fat fed ApoE^−/−^ mice, but in the absence of atherosclerotic lesions (Kitayama et al., 2007).

Our results indicate that brain inflammation is associated with chronic systemic inflammation, and an atherogenic diet further augments this process. In Paigen-fed ApoE^−/−^ mice an increase in T lymphocytes in the choroid plexus at the areas of granulocyte recruitment was seen. A recent report in experimental autoimmune encephalomyelitis highlights a key role of interleukin-17-producing T helper cells in recruiting immune cells into the choroid plexus (Reboldi et al., 2009). It is intriguing to speculate, therefore, that our data also highlight the possibility that such a process may occur as a result of chronic systemic inflammation alone. Alternatively, the brain inflammation may be driven by metabolic disturbances alone without the need for systemic inflammation.

Some ApoE^−/−^ mice fed a Paigen diet also displayed brain perivascular areas with focal lipid deposition and with microglial and vascular inflammation, similar to that seen in large peripheral blood vessels in these mice (Stoll and Bendszus, 2006; Zadelaar et al., 2007). Our data cannot confirm whether lipid deposition in the brain is a trigger of focal inflammatory changes or only a consequence of an ongoing inflammatory response. Nevertheless, we show that atherogenic diet is associated with focal inflammatory changes in the brain of animals that develop systemic vascular disease.

In summary, we demonstrate that chronic systemic inflammatory diseases, which are primary risk factors for stroke, are associated with inflammatory changes in the brain of rodents and humans. Our data support the existence of a causal relationship between systemic inflammation and brain inflammation that may contribute to stroke and other neurological disorders. An augmented inflammatory environment in the brain of stroke-prone individuals could aggravate post-ischaemic damage if stroke occurs and further studies will address this issue. Our translational approach has shown that appropriate co-morbid animal models exist that replicate important aspects of the stroke-prone state in humans, and that these co-morbid models could help facilitate translation from experimental studies to the clinic by providing a more realistic pre-clinical setting for testing novel therapies.

## Conflict of interest

Prof. N. Rothwell is a non-executive director of AstraZeneca, but there was no involvement of the company in any of these studies.

## Figures and Tables

**Table 1:** Clinical study group characteristics.

	At risk subjects (*n* = 4)	Control participants (*n* = 4)
Mean age in years [range]	63 [58–72]	64 [58–68]
Sex M:F	3:1	1:3
Number of risk factors [range]	3–4	1–2
Mean CRP at screening [range]	9.15 [2.99–13.26]	0.76 [0.55–1.00]
Mean interleukin-6 at screening [range]	12.00 [1.98–33.40]	2.46 [1.10–3.61]
Mean CRP at PET [range]	11.93 [8.98–15.73]	1.56 [1.18–2.05]
Mean interleukin-6 at PET [range]	10.55 [3.70–25.00]	4.79 [1.00–8.08]

## Appendix A Supplementary data

Supplementary Fig. 1 [^18^F]DPA-714 uptake in the kidneys, lungs (top graph) and heart (myocardium and ventricles; bottom graph) of lean and corpulent (Cp) rats at 9, 12 and 15 months of age. There were no significant difference between lean and corpulent animals nor between ages within each strain (*P* > 0.05, Mann–Whitney test). Data are expressed as and mean ± SD.
