# Supplementary material for: Brain inflammation is induced by co-morbidities and risk factors for stroke
Source: Brain Behav Immun. 2011 Aug;25(6-4):1113–22. doi: 10.1016/j.bbi.2011.02.008 (PMC3145158; doi:10.1016/j.bbi.2011.02.008)
Supplement: Supplementary Fig. 1 — [18F]DPA-714 uptake in the kidneys, lungs (top graph) and heart (myocardium and ventricles; bottom graph) of lean and corpulent (Cp) rats at 9, 12 and 15 months of age. There were no significant difference between lean and corpulent animals nor between ages within each strain (P > 0.05, Mann–Whitney test). Data are expressed as and mean ± SD. [file mmc1.doc]

###
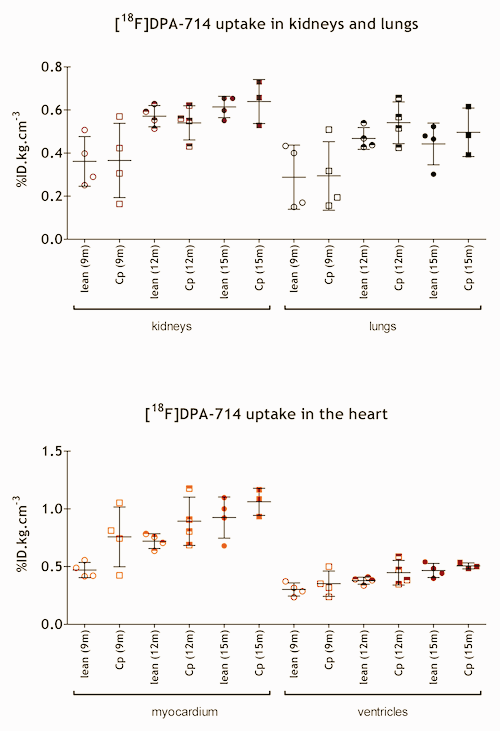


### Supplementary figure. [^18^F]DPA-714 uptake in the kidneys, lungs (top graph) and heart (myocardium and ventricles; bottom graph) of lean and corpulent (Cp) rats at 9, 12 and 15 months of age. There were no significant difference between lean and corpulent animals nor between ages within each strain (p>0.05, Mann-Whitney test). Data are expressed as and mean ± SD.
